# Mechanisms of tumor-associated macrophages in breast cancer and treatment strategy

**DOI:** 10.3389/fimmu.2025.1560393

**Published:** 2025-02-28

**Authors:** Hong Jin, Xinyue Meng, Jianwei Feng

**Affiliations:** Department of Ultrasound, Shengjing Hospital of China Medical University, Shenyang, Liaoning, China

**Keywords:** breast cancer, macrophages, TAMs, tumor microenvironment, HER2, treatment

## Abstract

Breast cancer (BC) is the most common cancer in women and a leading cause of cancer-related mortality. Despite advances in screening and treatment, outcomes for advanced or recurrent BC remain poor, highlighting the need for new strategies. Recent research emphasizes the tumor microenvironment (TME), particularly tumor-associated macrophages (TAMs), as key drivers of tumor growth, metastasis, and resistance to therapy. The presence of M2-like TAMs in the TME promotes immune evasion and tumor progression across BC subtypes. This review summarizes TAMs classification, their role in BC, and emerging therapies targeting TAMs, including depletion, inhibition of recruitment, and reprogramming from pro-tumoral M2 to anti-tumoral M1 phenotypes. Targeting TAMs offers a promising strategy to improve BC treatment outcomes.

## 1 Introduction

Breast cancer (BC) stands as the most frequently diagnosed malignant neoplasm among women and remains the foremost contributor to cancer‐related fatalities in the female population (1). Despite continuous refinements in screening approaches and therapeutic regimens, the prognosis for individuals with advanced or treatment‐resistant recurrent BC has fallen short of expectations. Developing more effective interventions aimed at decreasing relapse and improving clinical outcomes thus remains a critical imperative.

Recent investigations underscore the strong relationship between tumor and the tumor microenvironment (TME) (2–5). The TME denotes a sophisticated niche encompassing cells, signaling molecules, and the local physicochemical context, all of which modulate tumor cell proliferation (6), dissemination, progression (7–9), and responses to treatment (10–13).Within this specialized milieu, a variety of components—such as blood vessels, fibroblasts, neurons, inflammatory mediators, and immune populations including T cells, B cells, neutrophils, mast cells, and tumor‐associated macrophages (TAMs)—coexist around malignant cells (14, 15). Numerous stromal factors and cellular elements in the TME contribute to BC pathogenesis. Among these, TAMs represent a pivotal fraction of the microenvironment (16). By interacting with diverse cell populations, TAMs facilitate immune evasion and promote tumor progression (17, 18), ultimately influencing the clinical outlook for BC patients (19). They exert profound impacts on tumor growth, invasiveness, and therapy response (20). This review primarily discusses the roles and mechanisms of TAMs in the initiation and advancement of various BC subtypes, and further discusses emerging strategies that target these macrophages.

## 2 Classifications of TAMs

Most TAMs arise from bone marrow–derived monocytes, although a fraction are tissue-resident macrophages that infiltrate tumors from adjacent tissues. Once macrophages exit the circulation, their notable plasticity allows them to adopt distinct functions under various local microenvironmental stimuli. Commonly, they are categorized into two major phenotypes: classically activated M1 and alternatively activated M2 (21–23). The M1 subset is driven by Toll-like receptor (TLR) signaling, helper T cell 1 (Th1) cytokines, and interferon (IFN)-γ, which facilitates antigen presentation, amplifies cytotoxic functions, and participates in inflammatory responses as well as tumor immunosuppression. On the other hand, M2 macrophages arise in response to cytokines such as interleukin (IL)-4, IL-10, IL-13, and transforming growth factor-β (TGF-β), thereby restraining antitumor immunity, promoting tumor-associated vasculature formation, and advancing disease progression (24, 25).

At each phase of tumor progression, M1 and M2 macrophages coexist in varying ratios. During the initial stage, macrophages commonly favor the M1 phenotype to foster antitumor effects. However, as malignancies advance, M2 macrophages become dominant, forming the principal TAM subset in the TME (26). Moreover, M2 macrophages can be categorized into M2a, M2b, M2c, and M2d subtypes. Inflammatory factors are pivotal in inflammatory diseases progression and significantly influence the efficacy of therapies (27–30). For instance, Helper T cell 2 (Th2) cytokines (e.g., IL-4 and IL-13) prompt macrophages to acquire the M2a state, while immune complex signaling and TLR activation generate M2b cells, and IL-10 drives macrophages toward the M2c subtype (31). Macrophage polarization is tied to specialized metabolic pathways involving glucose, lipids, and glutamine. Evidence indicates that heightened glucose metabolism in TAMs triggers the buildup of various tumor-related metabolites, which facilitate the shift from the M1 to the M2 phenotype by modulating gene expression and signaling pathways, thereby granting TAMs a robust tumor-supportive capacity (32). These findings highlight that steering macrophages toward the antitumor M1 phenotype and limiting their transition into the tumor-promoting M2 state may offer an effective avenue for cancer treatment. M2 subtypes involve in BC progression. For instance,M2a macrophages differentiated *in vitro* through IL-4/IL-13 stimulation enhance the migratory and invasive capabilities of BC cells more prominently compared to M2b or M2c subtypes (33). M2b macrophages, activated through immune complex signaling and TLR pathways, contribute to immune modulation and inflammation, which can enhance metastasis and resistance in HER2-positive BC (34). M2c macrophages, driven by IL-10, are involved in immune suppression and tissue remodeling, facilitating the immune evasion of tumor cells in TNBC and enhancing the metastatic potential of cancer cells (34). M2d macrophages, characterized by their role in promoting angiogenesis and immune tolerance, support tumor growth and are particularly relevant in TNBC and HER2-overexpressing BC (34, 35).

## 3 The role of TAM in the occurrence and development of BC

### 3.1 The role of TAM in luminal-type BC

Of the various BC subtypes, the luminal category—characterized by estrogen receptor (ER) and/or progesterone receptor (PR) positivity—predominates. These luminal cells generally respond well to endocrine therapy yet show diminished sensitivity to chemotherapy. M2-oriented TAMs produce CCI2, which recruits monocytes into the TME and activates the phosphatidylinositol 3 kinase/protein kinase B/mammalian target of rapamycin (PI3K/AKT/mTOR) pathway, thereby driving endocrine resistance and establishing an endocrine-resistant niche. Additionally, individuals with ER-positive tumors and elevated CCI2 levels experience notably shorter progression-free survival compared with those exhibiting lower CCI2 expression (36). In THP-1 cells, diminished CXCR4 expression profoundly suppresses the oncogenic MAPK pathway and curtails the migratory capacity of MCF-7 cells (37). Wang et al. (38) demonstrated that, following co-culture of TAMs with MCF-7 cells, secretion of bone morphogenetic protein 2 (BMP-2) by TAMs increased sharply, leading to pronounced microcalcification within BC cells and ultimately contributing to unfavorable patient outcomes.

### 3.2 The role of TAM in HER2-overexpressing BC

An estimated 15%–20% of patients with breast carcinoma exhibit HER2 amplification, which predisposes them to higher rates of brain metastasis than those lacking HER2 overexpression, thereby creating a significant therapeutic challenge for this subgroup. In cancers displaying elevated HER2, TAMs can modulate the response to trastuzumab and consequently influence prognosis (39). In a humanized mouse model with human breast tumors overexpressing HER2, TAMs have been shown to regulate the antitumor actions of pertuzumab and trastuzumab via antibody-dependent phagocytosis (40). Among the cytokines secreted by tumor-associated macrophages (TAMs), interleukin-8 (IL-8) plays a pivotal role. In locally advanced HER2-positive breast cancer, IL-8 undermines the efficacy of lapatinib by activating the SRC/STAT3/ERK1/2 signaling cascade, which in turn stimulates the EGFR pathway. This interaction contributes to adverse clinical outcomes and the development of therapeutic resistance (41). Furthermore, matrix metalloproteinase 11 (MMP11) derived from macrophages promotes the migration of HER2^+^ tumor cells through the CCL2–CCR2 axis. Its pro-oncogenic influence may arise from inducing immunosuppression via upregulation of PD-L1 expression in BC cells (42).

### 3.3 The role of TAMs in TNBC

Triple-negative breast cancer (TNBC) exhibits poor pathological differentiation, marked invasiveness, and frequent recurrence and metastasis. Moreover, it remains largely unresponsive to endocrine and targeted therapies, resulting in a more adverse prognosis than that of other BC subtypes (43). Within TNBC lesions, TAMs are abundantly recruited and correlate with unfavorable clinical outcomes (44). Hypoxia in the tumor microenvironment is crucial for driving metastatic progression. In *in vitro* experiments, when oxygen availability is limited, TAMs secrete the pro-tumor metastasis factor CCL22, thereby enhancing the metastatic potential of MDA-MB-231 cells (45). Furthermore, Zhang et al. (46) revealed that in M2-type TAMs, the Yes-associated protein 1 (YAP1) modulates the monocyte chemoattractant protein 1 (MCP-1)/CCR2 pathway, leading to increased metastasis of MDA-MB-231 and BT549 cells.

TAMs encourage neoplastic cell proliferation by secreting bioactive molecules (47). Through lipocalin 2 (Lcn2) secretion, TAMs augment TNBC cell proliferation and motility. Additionally, Lcn2 antagonists impede the expansion and migration of TNBC cells (48). Among the central chemokines released by TAMs is C-X-C motif chemokine ligand 8 (CXCL8). While it may not substantially alter the proliferative ability of mammary carcinoma cells, CXCL8 heightens the invasiveness and miration of both murine and human breast tumors, triggers epithelial–mesenchymal transition (EMT), and supports the self-renewal capacity of BC stem cells (BCSCs) (49). In addition, IL-10 produced by M2-subtype TAMs enhances the Janus kinase 2 (JAK2)/STAT3 cascade, which fosters proliferation, invasion, and migration of MDA-MB-231 cells while curtailing apoptotic processes (50). Furthermore, TAM-produced VEGF-C supports vascular normalization in breast malignancies, reducing pulmonary dissemination of tumor cells yet elevating lymph node metastases (51).

Cytokines released by TAMs, exemplified by interleukin-6, promote the expansion of BCSCs through the STAT3 pathway. This cascade also upregulates essential transcription factors, such as sex-determining region Y box protein 2 (SOX2), octamer-binding transcription factor 3/4 (OCT3/4), and the Nanog homeobox (NANOG), thereby affecting the proliferation, metastatic competence, and neovascularization of 4T1 cells (52). Meanwhile, M2-skewed macrophages secrete vascular endothelial growth factor, triggering elevated expression of prostate cancer-associated transcript 6 (PCAT6), which in turn engages the VEGFR/AKT/mTOR axis, facilitating angiogenesis in TNBC (53). Cheng et al. (54) identified substantial B7-H3 expression within TAMs in patients with triple-negative breast tumors, noting that B7-H3-overexpressing TAMs display potent pro-angiogenic effects, impede T-cell infiltration, and promote metastatic dissemination. IL-17A promotes OPN secretion by tumor cells, activating LYVE-1/JNK/c-Jun signaling to expand immunosuppressive LYVE-1^+^ macrophages (55).

Exosomes secreted by TAMs also contribute to malignant cell dissemination. In 4T1 cells, uptake of these vesicles elevates miR-223-3p, which drives pulmonary metastasis by targeting chromobox homolog 5 (CBX5) (56). Macrophage-derived exosomes promote BC cell proliferation, invasiveness, and metastatic potential both *in vitro* and *in vivo* by activating the STAT3 signaling pathway and regulating key target genes, including CyclinD1, MMP2, and MMP9 (57, 58). Moreover, M2 macrophage-derived exosomes can deliver miR-92b into MDA-MB-231 cells, where this microRNA promotes autophagy in TNBC cells by suppressing phosphatase and tensin homolog (PTEN) (59). Collectively, these findings indicate that TAMs critically affect TNBC progression via the release of cytokines, chemokines, and exosomes. TGF-β1 originating from TAMs modulates hepatic leukemia factor (HLF), recognized as a novel oncogenic mediator in TNBC. Consequently, this factor enhances ferroptosis resistance by inducing γ-glutamyl transferase 1 (γ-GGT1), thereby supporting tumor cell proliferation and elevating drug resistance (18).

In BRCA-associated TNBC, therapy with olaparib stimulates macrophage maturation and bolsters antitumor mechanisms, although its effect proves short-lived. Subsequent immunosuppressive cascades emerge, marked by upregulated PD-L1 and colony stimulating factor 1 (CSF1) receptor, alongside a strong dependence on lipid metabolism. TAMs relying on lipid-based pathways demonstrate immunosuppressive properties, thereby constraining the efficacy of PARP inhibitors and contributing to resistance (60).

**Figure 1:**
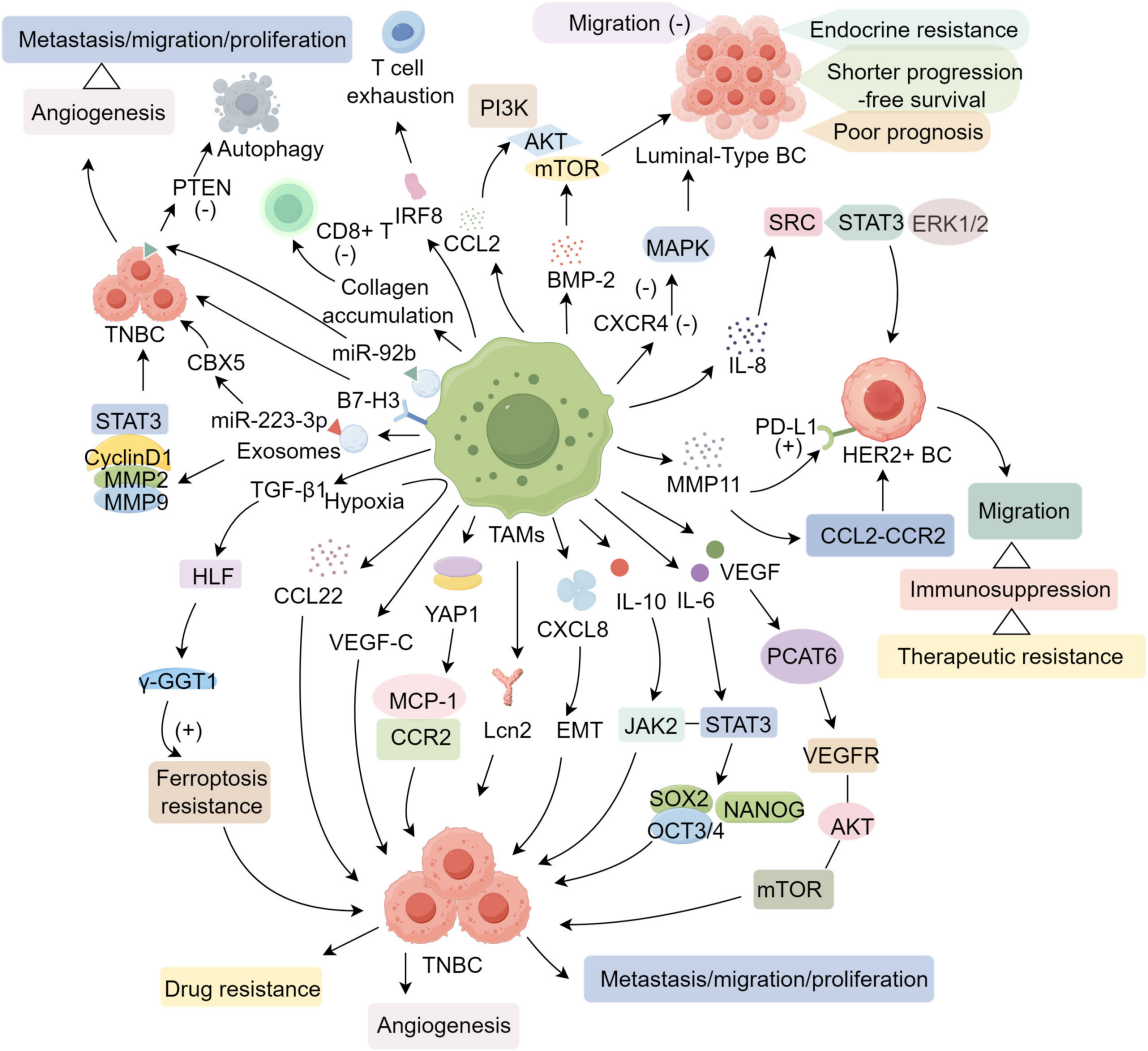
The mechanisms of TAMs in BC.

Cross talk between TAMs and other immune cells contribute to immune evasion and progression in BC. Interaction between IL-15Rα^+^ TAMs and BC cells reduces CD8^+^ T cell recruitment in TME (61). Besides, TAMs inhibit the activity of CD8^+^ T cells by promoting collagen accumulation and altering the metabolic environment within BC (62). TAMs that express the transcription factor IRF8 play a significant role in inducing T cell exhaustion in the TME of BC (63). The density of tumor-associated neutrophils (TANs) was correlated with the presence of CD163^+^ M2-like TAMs. M1/M2 TAM ratio was elevated, TANs served as a negative prognostic indicator in BC (64). (**Figure 1**).

## 4 Targeting TAM therapy

### 4.1 Reducing TAMs

TAMs are pivotal in facilitating tumor cell growth, invasion, metastasis, and the formation of new blood vessels. Targeting and eliminating TAMs along with their precursor cells represents a significant strategy in anti-cancer therapies. Bisphosphonates (BP) are extensively utilized for managing patients with bone metastases. Evidence from clinical trials indicates that BP medications enhance the survival outcomes of BC patients. Additionally, both experimental models and clinical studies have shown that TAMs uptake BP, leading to their apoptosis or impaired functionality, thereby inhibiting tumor progression (65, 66). Chemotherapeutic drugs like docetaxel also suppress TAMs to manage tumor advancement. In a mouse model of orthotopic BC using the 4T1 cell line, the combined intraperitoneal administration of zoledronic acid (ZA) and docetaxel resulted in a significantly greater reduction in tumor growth and lung metastasis compared to ZA alone (67). Furthermore, doxycycline effectively depletes CD68^+^ TAMs. In the PyMT-Maclow BC mouse model, treatment with doxycycline decreased TAMs surrounding the tumor margins by 43%, thereby slowing tumor growth, reducing angiogenesis, and lowering the expression levels of genes that promote blood vessel formation (68). Recently, electroacupuncture treatment was shown to restore normal vascular function in TNBC through the reduction of glyoxalase-1 levels, which facilitated the polarization of TAMs toward the M1 phenotype (69).

### 4.2 Inhibition of TAM recruitment

Chemokines, cytokines, and complement components drive TAM recruitment in BC. CCL2 binding to CCR2 on monocytes facilitates TAM infiltration, making the CCL2/CCR2 pathway a key therapeutic target. Chitosan-DNA nanoparticles/siRNA targeting CCR2 (CNP/siCCR2) nanoparticles inhibit TAM recruitment, reduce tumor immunosuppression, and enhance chemotherapy efficacy in BC models (70). Elevated CCL2 and CCL5 in ER^+^ BC lead to macrophage infiltration and angiogenesis, which are reduced by anti-CCL2/CCL5 therapies (71).

CSF1 is crucial for macrophage proliferation and differentiation, with high levels linked to poor BC prognosis (72). Targeting CSF1 expression effectively diminishes TAM infiltration and enhances the chemosensitivity of BC. Pignatelli et al. (73) demonstrated that antibodies directed against the CSF1R impede the trans-endothelial migration of BC cells, proposing CSF1 as a potential therapeutic target for metastasis across multiple bBC subtypes. In murine BC models, the inhibition of CSF1/CSF1R signaling through either CSF1 monoclonal antibodies or the CSF1R tyrosine kinase inhibitor PLX3397 results in the depletion of TAMs and significantly postpones tumor recurrence following radiotherapy (74).

Angiogenesis is fundamental to both the initiation and progression of BC, with Vascular Endothelial Growth Factor (VEGF) being the principal angiogenic factor secreted by TAMs. Consequently, the VEGF/VEGFR2 axis has become a focal point of research; however, challenges such as therapeutic resistance, tumor evasion, recurrence, and potential harm to normal tissues present significant obstacles. Song et al. (75) reported that the administration of specific small interfering RNAs targeting VEGF and Placenta Growth Factor (PIGF) via nanoparticles to M2-polarized TAMs and BC cells as an immunotherapeutic approach markedly inhibits BC growth and pulmonary metastasis.

### 4.3 Reprogramming TAMs

M1 macrophages exhibit tumor-suppressive properties, whereas TAMs, which share characteristics with M2 macrophages, promote tumor progression. Current research is focused on reprogramming macrophages toward anti-tumor phenotypes. A study by Guerriero et al. demonstrated that the class IIa histone deacetylase inhibitor (HDACi) TMP195 effectively reduced tumor-promoting macrophages while increasing anti-tumor macrophages in murine models. This shift led to a reduction in tumor growth and lung metastasis. Furthermore, TMP195, when combined with chemotherapy or immune checkpoint blockade, significantly enhanced the anti-tumor response, suggesting its potential as an adjuvant in cancer therapy (76). Emerging small-molecule HDAC inhibitors, such as entinostat, when used in combination with immune checkpoint inhibitors (ICIs), have been found to facilitate the transition of M2-type TAMs to the M1 phenotype, concurrently suppressing NF-κB and STAT3 signaling pathways, thereby curbing tumor progression (77). The chemotherapeutic agent cabazitaxel has the capacity to restore TAM-mediated programmed cell removal (prCR) in BC by promoting macrophage polarization toward the M1 type, activating the TLR/NF-κB axis, and enhancing the secretion of pro-inflammatory cytokines (78).

TLRs, essential pathogen recognition receptors on immune cells, play a pivotal role in immune modulation. In a murine model, the administration of a TLR3 ligand facilitated the transformation of M2-like TAMs back to the M1 phenotype through the IFN-α/β signaling pathway, thereby suppressing tumor growth (79). Lei et al. (80) found that the FAPα enzyme-activated vinca alkaloid prodrug ZGP-DAVLBH, a vascular disrupting agent, enhanced the secretion of granulocyte-macrophage colony-stimulating factor (GM-CSF) in both tumor tissues and the serum of tumor-bearing mice. This led to a phenotypic shift of TAMs from M2 to M1 and induced apoptosis in the doxorubicin-resistant BC cell line MCF-7/ADR. Additionally, Tasquinimod, an oral quinoline-3-carboxamide, was shown to convert immunosuppressive M2-type TAMs into pro-inflammatory M1-type TAMs in a 4T1 orthotopic BC model, resulting in inhibited tumor growth and reduced pulmonary metastasis (81).

Nanotechnology offers precise drug delivery to specific cellular targets. Targeting TAMs through nanocarriers can induce their polarization to the M1 phenotype, thereby modulating their phagocytic activity and ultimately regulating BC growth, metastasis, and invasion. Examples include hybrid nanoparticles encapsulating paclitaxel and baicalin (HPoNPC) (82), macrophage membrane-coated nanoparticles loaded with the TGF-β R1 inhibitor SD208 (Mφ-SDNP) (83), and organic polymer nanoparticles (CPTH) (84). These nanostructures effectively promote the shift of TAMs from M2 to M1, specifically target BC cells, and exhibit potent anti-tumor effects. Extracellular vesicles from Platycodon grandiflorum (PGEVs) promote TAM repolarization to M1, inhibiting TNBC progression (85). Recent strategy to enhance tumor oxygenation, converts immunosuppressive M2 to antitumor M1 macrophages offering promising in overcoming BC drug resistance (86). Matairesinol (MAT), a plant lignan, exhibits anticancer and immunomodulatory effects. It reduces M2a and M2d macrophage viability, promoting their shift to the M1 phenotype. Conditioned medium from MAT-treated macrophages induces apoptosis in TNBC cells, highlighting MAT’s potential as a novel anti-TNBC therapeutic (87).

## 5 Conclusion

TAMs are integral components of the BC TME, exerting profound influences on tumor progression, metastasis, and resistance to therapy. The dualistic nature of TAMs, characterized by their ability to adopt either pro-inflammatory M1 or anti-inflammatory M2 phenotypes, underscores their pivotal role in shaping the immune landscape of BC. The dominance of M2-like TAMs in various BC subtypes, including luminal, HER2-positive, and triple-negative forms, is closely associated with unfavorable clinical outcomes and reduced therapeutic efficacy. Therapeutic strategies targeting TAMs—ranging from their depletion and inhibition of recruitment to the reprogramming of their phenotypic states—offer multifaceted approaches to disrupt the pro-tumoral activities mediated by these cells. Advances in pharmacological agents, molecular inhibitors, and nanotechnology-based delivery systems have demonstrated potential in shifting TAMs toward a tumor-suppressive M1 phenotype, thereby enhancing the effectiveness of existing treatments and mitigating metastatic spread. Continued exploration of the intricate interactions between TAMs and BC cells is essential for the development of more effective, TAM-targeted therapies. Ultimately, integrating TAM modulation into comprehensive BC treatment regimens holds significant promise for improving patient prognoses and overcoming current therapeutic limitations.
